# A Comparison of Depressive Symptom Presentation in Adolescent Type 1 Diabetes and Pediatric Primary Care Samples

**DOI:** 10.1155/2023/5597133

**Published:** 2023-08-23

**Authors:** Nasreen Bahreman, Mary S. Dietrich, Sarah Jaser, Terrah Akard Foster, Shelagh Mulvaney

**Affiliations:** ^1^Towson University, Towson, USA; ^2^School of Nursing, Vanderbilt University, Nashville, USA; ^3^Department of Biostatistics, Vanderbilt University Medical Center, Nashville, USA; ^4^Department of Pediatrics, Vanderbilt University Medical Center, Nashville, USA; ^5^Department of Biomedical Informatics, Vanderbilt University Medical Center, Nashville, USA

## Abstract

**Background:**

Depression is a common comorbidity in adolescents with type 1 diabetes (T1D). It is unclear if patterns of responses from questionnaires used to screen for depressive symptoms are influenced by the burden of living with T1D and/or the consequences of hyperglycemia. Based on this gap in the adolescent research, we sought to identify potential differences in depression screening response patterns between adolescents with and without T1D and relate response patterns with glycemic outcomes.

**Methods:**

Using a retrospective case–control design, we analyzed electronic health records for age, sex, and race-matched adolescents 13–18 years of age from a pediatric diabetes clinic (*n* = 477) and a pediatric primary care clinic (*n* = 477) in the United States. Adolescents in both settings were screened for depressive symptoms during the same time period using the Patient Health Questionnaire-9 (PHQ-9).

**Results:**

Participant demographics for matched characteristics were: 53.5% male, 71.7% White, median age 13.0 (interquartile range = 13.0, 14.0). After controlling for type of insurance, adolescents with T1D were more likely to have higher total PHQ-9 scores (odds ratio (OR) = 1.51, 95% CI = 1.17, 1.98, *p* = 0.002) and higher somatic subscores (OR = 1.57, 95% CI = 1.20, 2.05, *p* = 0.001) compared to the primary care sample. The pattern of item endorsement greater than “not at all” indicated that adolescents with T1D were more likely to have higher values for somatic items such as “trouble falling asleep” and “feeling tired” than those in the primary care sample. Item-total correlations and Cronbach's *α* indicated that all items were contributing to the overall score in the same manner in each group.

**Conclusions:**

Symptom endorsement for sleep and fatigue were higher for adolescents with T1D and without T1D. Study results support the need for further examination of the origins of somatic symptoms in T1D and for an additional examination of the specificity of depression screening instruments used in routine pediatric diabetes care.

## 1. Introduction

Adolescence is a developmental period characterized by physical, psychosocial, and cognitive changes [1]. Adolescence is also a time of increased incidence of depression. In 2020, an estimated 4.1 million adolescents in the general population of the United States reported at least one episode of major depression, which represented 17% of individuals between 12 and 17 years of age [2]. For adolescents with type 1 diabetes (T1D), challenges may be magnified during this developmental period due to the demands of managing T1D. Routine depression screening in primary care and diabetes care is recommended for adolescents [3–6]. Symptoms of depression are twice as likely to be present in adults living with T1D compared to persons without T1D [7]. Rates of elevated symptoms of depression (that use various measures) in adolescent T1D range from 6% to 30% [8–12]. Depression is a noted contributor to poor glycemic outcomes in adults [13]. However, studies in adults and adolescents living with T1D and type 2 diabetes (T2D) have found variable relationships between depression screening instruments and glycemic outcomes [14].

There is some indication that, in addition to differing screening instruments used across studies, variability in depression-blood glucose relationships may be due to the nature of the phenomenon being assessed by the screeners. Some depression screening items may be endorsed more frequently as symptoms of hyperglycemia or the burden of living with T1D [15]. Relatedly, limited research has identified the somatic symptoms of depression as more positively related to glycemic outcomes or even in the opposite direction compared to the cognitive–affective symptoms of depression in adults [15, 16]. Similar findings of differential somatic versus affective or cognitive symptom presentation and relation to HbA1c may exist in adolescents [17]. Depression measurement issues have been identified in other chronic illness populations due to the overlap in symptom presentation, such as in people with multiple sclerosis related to fatigue and concentration [18, 19] and poor appetite in youth with inflammatory bowel disease [20]. In people with T1D, some of the manifestations of hyperglycemia may present as similar to depressive symptoms, including sleep disturbance [21, 22], trouble with concentration [23], fatigue, and feelings of failure and shame related to poor glycemic outcomes [24–26]. If adolescents endorse depression screening items due to similarity with the effects of hyperglycemia, this could result in inaccurate causal inferences and inappropriate treatment of symptoms.

Research is not clear in this area, as McDade-Montez and Watson [27] found no differences in symptom endorsement patterns in a multisample confirmatory factor analysis for adults with either T1D or T2D and without diabetes. While somatic screening items have been differentially associated with glycemic outcomes [17, 21, 22], little research has directly compared symptom patterns in youth with and without T1D [28]. Based on this gap in the adolescent research and elevated somatic symptoms in depression screening in other pediatric conditions, we sought to identify potential differences in depression screening response patterns between adolescents with and without T1D and relate response patterns to glycemic outcomes in T1D. We hypothesized that somatic symptoms of depression would be elevated in adolescents with T1D compared to adolescents without T1D.

## 2. Methods

### 2.1. Study Design and Participants

This retrospective case–control study analyzed existing data from completed Patient Health Questionnaire-9 (PHQ-9) measures administered for depression screening and saved in the electronic health records (EHR) of adolescents (13–18 years) attending two clinics affiliated with a large academic medical center. The study sample included adolescents with T1D who were screened in the diabetes clinic at least once between February, 2016 and December, 2020 and adolescents from the pediatric primary care clinic without diabetes (primary care group) with at least one depression screening during that same time period. After matching the two groups for age, sex, and race, the merged data set included 477 matched pairs of adolescents.

Subjects were excluded if they had a diagnosis of a serious mental illness such as schizophrenia, had missing responses to any PHQ-9 items, or had a diagnosis of T2D based on ICD-10 codes. Subjects were excluded from the primary care group if they had any diagnosis of diabetes. Researchers deidentified data and assigned a unique subject identification number to each subject. For age matching, a period of ±3 months was used as the criterion range for a match.

### 2.2. Procedures

Researchers obtained Institutional Review Board approval before the study activity began. Both settings used the PHQ-9 for routine depression screening. Adolescents with T1D completed a depression screening every 6 months during routine clinic visits using a tablet, and pediatric primary care patients completed screening using a paper version once per year. Only the first depression screening available for each subject was used for analyses.

### 2.3. Measures

The PHQ-9 is a 9-item self-report instrument developed from the Primary Care Evaluation of Mental Disorders instrument to screen for the presence and severity of depressive symptoms over the last 2 weeks [29]. The PHQ-9 is written at a fifth-grade reading level. Items are rated on a 4-point Likert scale (0 = not at all, 3 = nearly every day). The total is the sum of the items with a possible total score of 0–27. Scores ≥10 (moderate or greater) are considered a positive screen [30]. The content validity of the measure is supported by the nine items that are directly derived from the symptoms of major depressive disorder identified in the DSM-5 [31]. Internal consistency (Cronbach's *α*) of the PHQ-9 has been reported as 0.85–0.89 in previous studies [8, 32]. In the current sample, the internal consistency of the full PHQ-9 for the T1D group was *α* = 0.83, and for the primary care group, *α* = 0.82. Based on previous research examining somatic versus affective depression symptoms, four PHQ-9 items were analyzed as part of a somatic subscale (fall asleep, appetite, feeling tired, moving slowly), and five were analyzed as cognitive–affective (feeling down, little interest, feeling bad about self, trouble concentrating, thoughts of self-harm) [15, 17, 33]. Cronbach's *α*s were adequate for the somatic (T1D = 0.70, primary care = 0.71) and the cognitive–affective subscores (T1D = 0.72, primary care = 0.73) Adolescent age, sex, type of insurance (private, public), race and ethnicity were obtained from the EHR and are obtained via parent report in clinical encounters.

### 2.4. Data Analyses

Study groups were matched on age, race, and gender based on research documenting relationships between those demographic variables and depressive symptoms in adolescents and to match generally on developmental status using age [34–36]. The PHQ-9 total scores were extremely positively skewed, so we included median and interquartile ranges (IQRs) and frequencies of PHQ-9 categories (which range from minimal to severe) to summarize the two groups. Assuming that previously reported mean and SDs are from normally distributed samples, our median and IQR values can be compared to those reports for generalizability purposes. In addition, crosstabulations of individual item responses (0–3) by study group were used to generate summaries of those responses using counts and percentages. Previous research has documented higher depressive symptoms in those youth with poor glycemic outcomes [17]. We examined the levels of depressive symptoms for the individuals in the T1D group with HbA1c above and below the median of the sample using *t*-tests of the log-transformed to normal symptom distributions. Given the confounding of the type of insurance with the T1D group, the type of insurance was included as a covariate in all analyses.

Due to the extremely skewed distributions and ordinal-level scaling of the item responses, ordinal logistic regression analyses were used to test the effects of group membership (T1D versus primary care) on the PHQ-9 total, somatic subscore, cognitive–affective subscore, and item-level scores. The proportional-odds assumption underlying those types of models was tested. That assumption was met for the total, somatic, cognitive–affective models (*p* > 0.20) and for all but one of the individual item models (*p* > 0.05; “poor appetite,” *p* = 0.025). To compare rates of item responses between groups, item responses of “0” were defined as “not endorsed” and responses of “1–3” were defined as “endorsed.” To compare the relative contribution of each PHQ-9 item to the total scores between groups, the corrected item-total correlation of each item with the total score was evaluated. Analyses were conducted using *IBM SPSS Statistics* (Version 28.0, Released 2021. Armonk, NY: IBM Corp) and *Stata* (Version 15. College Station, TX: StatsCorp LLC). An *α* of .05 (*p* < 0.05) was used for interpretations of statistical significance.

## 3. Results

Participant demographics for the matched characteristics were: 53.5% male, median age 13.0 (IQR = 13.0, 14.0), 71.7% White, 19.5% Black, 0.4% Asian, and 8.4% unknown. Demographic variables that were unmatched included insurance type (private vs. public) and ethnicity (Hispanic vs. Non-Hispanic). The two cohorts were different in both characteristics, with 57.4% of the adolescents with T1D having private insurance compared to 10.9% of primary care participants. The large imbalance in proportions of public vs. private insurance between the groups precluded matching on that variable. A higher percentage of adolescents in the primary care group identified as Hispanic compared to those with T1D (44.1% vs. 5.7%; *p* < 0.001). There was a confounding of insurance type with ethnicity, with 95.6% of those with private insurance identifying as non-Hispanic compared to 64.2% of those with public insurance. Given that there were relatively more cases missing information for ethnicity, insurance type was used as a covariate in all analyses.

Given that data collection spanned a period of time before and during the COVID-19 pandemic, we assessed the differences in PHQ-9 scores for the samples across those two time periods. There was no difference between the PHQ-9 scores before (defined before March, 2020) or after that date during the COVID-19 restrictions.

### 3.1. T1D Group

The adolescents with diabetes had a median HbA1c of 8.7 (IQR 7.5 10.7) at the time of screening. The PHQ-9 total and subscores were very similar for the adolescents with HbA1c above and below the median value (Cohen's *d*: 0.02, 95% CI = −0.16, 0.20 (total); <0.01, 95% CI = −0.18, 0.18 (somatic); 0.13, 95% CI = −0.05, .32 (cognitive–affective); all *p* > 0.10).

### 3.2. Comparison of T1D and Primary Care Groups

The mean PHQ-9 for the T1D group was 3.0 (SD 2.5), and for the primary care group was 2.7 (SD 2.5). The median PHQ-9 total scores and IQR were the same for both groups (median 2.0, IQR 0.0, 5.0) and not statistically significant (*p* = 0.076). However, there was a higher percentage of adolescents in the moderate or above PHQ-9 categories (score ≥10) in the T1D sample (10.5%) than in the primary care sample (7.9%, Table 1).

Adolescents in the T1D group were more likely to have higher somatic scores than those in the primary care group (odds ratio (OR) = 1.57, 95% CI = 1.20, 2.05, *p* = 0.001) but not cognitive–affective scores (OR = 1.23, 95% CI = 0.94, 1.62, *p* = 0.136). Summaries and comparisons of PHQ-9 item-level response patterns for T1D and primary care groups are shown in Table 2.

Consistent with the observed differences in the somatic subscale scores, item response distributions showing the greatest differences between the groups were in that domain. Adolescents with T1D were more likely to have higher values for “trouble falling asleep” (OR = 1.75, *p* < 0.001) and “feeling tired” (OR = 1.36, *p* = 0.039). Given the skewed distributions of item responses shown, the median values for all items for both groups were 0.0, with the 75th quartile values for both groups also being 0.0 for 5 of the nine items. The 75th quartile value was 1.0 for both groups for the following items: “Little interest/pleasure,” “Trouble falling asleep,” “Feeling tired,” and “Trouble concentrating.”

As shown in Table 3, the Cronbach's *α* values for the total scores in both T1D and primary care groups were almost identical, as were the patterns of the corrected item to total score correlations. Those values ranged from 0.47 (“thoughts of self-harm”) to 0.62 (“down and depressed”) in the T1D sample and from 0.44 (“moving or talking slowly”) to 0.64 (“feeling bad about self”) in the primary care group. The amount of change in Cronbach's *α*, if an item is deleted, indicates how much the given item response value is contributing to the total score. If the Cronbach's *α* is greater with the item is deleted, then the item is not loading well with the other items. If Cronbach's *α* decreases when the item deleted, the item is contributing to the total score. Within both of the groups, none of the items revealed an increase in Cronbach's *α* if they were deleted, and the patterns of the reductions in Cronbach's *α* for each item across the groups were very similar.

## 4. Discussion

The goals of this study were to compare patterns of depressive symptoms in adolescents with T1D to matched adolescents in primary care and to examine patterns of responses that may align with either somatic and/or cognitive–affective subscores and with glycemic outcomes. To our knowledge, this is the first study to examine the severity of depressive symptoms and symptom endorsement patterns in adolescents with T1D and an age-, sex-, and race-matched cohort of adolescents from a primary care setting. The retrospective study included large samples of T1D and primary care subjects who completed routine depression screening at the same medical center during the same time period. Both groups indicated levels of depressive symptoms that are within the typical ranges of prevalence documented in the United States [8, 37]. We did not find differences in levels of depressive symptoms for adolescents that had poor glycemic outcomes. Overall there were no differences between the groups in median total PHQ-9 scores or on the cognitive–affective subscores. The groups were different on the level of somatic symptoms overall. Adolescents with T1D had higher somatic symptom scores.

Item-level analyses showed symptom presentations that were largely similar, with two somatic symptom exceptions. The likelihood of adolescents with T1D endorsing trouble falling asleep and experiencing fatigue was greater than that of the primary care sample. Reports of trouble falling asleep are consistent with depression and with research documenting sleep disturbance related to the use of diabetes technologies [38]. Sleep disturbance assessed by the PHQ-9 may be caused by alarms associated with diabetes devices or the awareness of a device on the body [39]. Sleep is increasingly a treatment target in diabetes care in relation to the use of diabetes technologies [40]. Brief and feasible interventions, including cognitive behavior therapy and coaching, are efficacious in improving the initiation and maintenance of sleep and simultaneously address symptoms of depression in adolescents [41–43]. Structured education and support from other device users have also been successfully utilized to reduce barriers to diabetes device use, such as sleep disruption, in this population [44]. Similarly, the symptom of fatigue has more than one potential cause. It is consistent with depression and may also be caused by hyperglycemia [45, 46]. Although the physiological relationship between fatigue and hyperglycemia may not be modifiable, recent developments in assessment methods, such as ecological momentary assessment, could provide more specific individual insights between fatigue as caused by blood glucose excursions versus a general feeling of fatigue as related to depression.

Item-level comparisons did not reveal differences in item-to-total correlations in the T1D and primary care cohorts, indicating that the items were contributing similarly to the total scores for each group. Although there were no item-level differences in thoughts of harm to self, recent research has reported that depression symptom screening instruments may not be adequately sensitive in detecting the risk for suicide in adolescents and specifically adolescent T1D [47, 48]. Some authors have recommended downwardly adjusted cutoff scores, including specific suicidality measures or using a brief interview with the screener [47, 49–51]. Additional validation of screening tools against diagnostic gold-standard methods in depression is needed in this population, as is the identification of clinically significant change metrics for widely used assessment tools in pediatric diabetes.

This study represents the first step in the documentation of differences in item response patterns between adolescents with and without T1D. We note several limitations. We were limited to the data available in the EHR and did not have access to the duration of T1D. The results document somatic symptom differences between T1D and primary care samples but do not provide insights regarding the causes or mechanisms of these differences. This overlap in symptom presentation should be teased apart in future research. Prospective research should utilize mixed methods, and integrate cognitive interviews, to identify subjective perceptions and causal attributions of adolescents when responding to screening questions. Diabetes professionals may need caution in the interpretation of somatic depressive symptoms, particularly when an elevated PHQ-9 score is driven by high somatic item scores. Follow-up questions regarding the nature of the symptoms may be useful, specifically when symptoms related to sleep or fatigue impact the overall score. A brief discussion and exploration of those item scores with individuals could provide context and additional insights regarding next steps and referrals for further diagnostic assessment.

Although the samples were matched on age, sex, and race, a much greater portion of adolescents from the pediatric primary care clinic had public insurance. Insurance status may be viewed as a proxy variable for family socioeconomic challenges, and depressive symptoms have been associated with lower family income in T1D [52]. Although analyses were adjusted, the low percentage of adolescents with private insurance coverage in the primary care sample precluded matching on that variable. Children with T1D from families that experience low family income, housing instability, or food insecurity are more likely to have poor glycemic outcomes [53–55]. Future quasi-experimental research should match comparison groups on insurance or other relevant socioeconomic variables and social determinants of health when possible. More importantly, researchers should explicitly include social determinants in estimating factors that impact suboptimal outcomes in adolescent T1D [54, 56].

## Figures and Tables

**Table 1:** PHQ-9 score categories for the T1D and primary care groups.

PHQ-9 score category (range)	T1D	Primary care
	*n* (%)	*n* (%)
Minimal (0–4)	329 (69.0)	353 (74.0)
Mild (5–9)	98 (20.5)	86 (18.0)
Moderate (10–14)	39 (8.2)	31 (6.5)
Moderately severe (15–19)	6 (1.3)	4 (0.8)
Severe (≥20)	5 (1.0)	3 (0.6)

**Table 2:** PHQ-9 item endorsement rates by group and odds ratios for logistic regression (*N* = 954).

PHQ-9 item	*Not at all n* (%)	*Several days n* (%)	*>½ days n* (*%*)	*Every day n* (*%*)	*OR* (95% CI)^a^	*p* ^a^
Feeling down depressed					1.27 (0.88, 1.81)	0.189
T1D	363 (76.1)	85 (17.8)	19 (4.0)	10 (2.1)		
Primary care	384 (80.5)	67 (14.0)	18 (3.8)	8 (1.7)		
Little interest pleasure					1.21 (0.88, 1.66)	0.223
T1D	335 (70.2)	99 (20.8)	26 (5.5)	17 (3.6)		
Primary care	326 (68.3)	116 (24.3)	25 (5.2)	10 (2.1)		
Trouble falling asleep					1.75 (1.31, 2.34)	<0.001^*∗∗*^
T1D	258 (54.1)	116 (24.3)	53 (11.1)	50 (10.5)		
Primary care	290 (60.8)	108 (22.6)	41 (8.6)	38 (8.0)		
Poor appetite					1.21 (0.85, 1.72)	0.281
T1D	371 (77.8)	72 (15.1)	17 (3.6)	17 (3.6)		
Primary care	376 (78.8)	72 (15.1)	21 (4.4)	8 (1.7)		
Feeling tired					1.36 (1.01, 1.81)	0.039^*∗*^
T1D	269 (56.4)	151 (31.7)	30 (6.3)	27 (5.7)		
Primary care	291 (61.0)	134 (28.1)	32 (6.7)	20 (4.2)		
Feeling bad about self					1.06 (0.71, 1.56)	0.776
T1D	390 (81.8)	61 (12.8)	13 (2.7)	13 (2.7)		
Primary care	398 (83.4)	51 (10.7)	19 (4.0)	9 (1.9)		
Trouble concentrating					1.23 (0.90, 1.67)	0.193
T1D	323 (67.7)	83 (17.4)	44 (9.2)	27 (5.7)		
Primary care	326 (68.3)	93 (19.5)	37 (7.8)	21 (4.4)		
Moving/speaking slowly					1.45 (0.94, 2.24)	0.089
T1D	408 (85.5)	52 (10.9)	12 (2.5)	5 (1.0)		
Primary care	422 (88.5)	39 (8.2)	9 (1.9)	7 (1.5)		
Thoughts of self-harm					1.68 (0.88, 3.17)	0.113
T1D	446 (93.5)	24 (5.0)	4 (0.8)	3 (0.6)		
Primary care	456 (95.6)	17 (3.6)	1 (0.2)	3 (0.6)		

Values in cells are “*n* (%)” within groups; T1D: *N* = 477; Primary care: *N* = 477. ^a^Adjusted for insurance type;  ^*∗*^*p* < 0.05;  ^*∗∗*^*p* < 0.001.

**Table 3:** PHQ-9 corrected item–total correlations and Cronbach's *α* if item deleted.

Reliability (Cronbach's *α*)	T1D (*N* = 477)		Primary care (*N* = 477)	
	0.83		0.82	
Item	*r* ^a^	*α* ^b^	*r* ^a^	*α* ^b^
Down and depressed	0.62	0.80	0.63	0.80
Little interest or pleasure	0.53	0.81	0.50	0.81
Trouble falling asleep	0.59	0.81	0.58	0.80
Poor appetite	0.50	0.81	0.52	0.81
Fatigue	0.58	0.81	0.64	0.79
Feeling bad about self	0.58	0.81	0.56	0.80
Trouble concentrating	0.56	0.81	0.54	0.81
Moving or talking slowly	0.52	0.82	0.44	0.82
Thoughts of self-harm	0.47	0.82	0.45	0.82

*Note*. ^a^*r* represents corrected item–total correlation. ^b^Indicates internal consistency of remaining items if the selected item is deleted from the total score.

## Data Availability

The data use agreement does not support data sharing with third parties.
